# Markers of blood-brain barrier disruption increase early and persistently in COVID-19 patients with neurological manifestations

**DOI:** 10.3389/fimmu.2022.1070379

**Published:** 2022-12-15

**Authors:** Valentina Bonetto, Laura Pasetto, Ilaria Lisi, Marco Carbonara, Rosalia Zangari, Erica Ferrari, Veronica Punzi, Silvia Luotti, Nicola Bottino, Bruno Biagianti, Cristina Moglia, Giuseppe Fuda, Roberta Gualtierotti, Francesco Blasi, Ciro Canetta, Nicola Montano, Mauro Tettamanti, Giorgia Camera, Maria Grimoldi, Giulia Negro, Nicola Rifino, Andrea Calvo, Paolo Brambilla, Francesco Biroli, Alessandra Bandera, Alessandro Nobili, Nino Stocchetti, Maria Sessa, Elisa R. Zanier

**Affiliations:** ^1^Istituto di Ricerche Farmacologiche Mario Negri IRCCS, Milan, Italy; ^2^Fondazione IRCCS Ca’ Granda Ospedale Maggiore Policlinico, Milan, Italy; ^3^FROM Research Foundation, Papa Giovanni XXIII Hospital, Bergamo, Italy; ^4^Department of Pathophysiology and Transplantation, University of Milan, Milan, Italy; ^5^“Rita Levi Montalcini”, Department of Neuroscience, University of Turin, Turin, Italy; ^6^AOU Città della Salute e della Scienza Hospital, Turin, Italy; ^7^Department of Neurology, Papa Giovanni XXIII Hospital, ASST Papa Giovanni XXIII, Bergamo, Italy; ^8^Neurology Section, School of Medicine and Surgery, University of Milano-Bicocca, Monza, Italy; ^9^Division of Neurology, University of Milano-Bicocca, Milan, Italy

**Keywords:** COVID-19, neurological damages, blood-brain barrier, inflammation, blood biomarkers, critical care

## Abstract

**Background:**

Coronavirus disease 2019 (COVID-19) caused by SARS-CoV-2 infection is associated with disorders affecting the peripheral and the central nervous system. A high number of patients develop post-COVID-19 syndrome with the persistence of a large spectrum of symptoms, including neurological, beyond 4 weeks after infection. Several potential mechanisms in the acute phase have been hypothesized, including damage of the blood-brain-barrier (BBB). We tested weather markers of BBB damage in association with markers of brain injury and systemic inflammation may help in identifying a blood signature for disease severity and neurological complications.

**Methods:**

Blood biomarkers of BBB disruption (MMP-9, GFAP), neuronal damage (NFL) and systemic inflammation (PPIA, IL-10, TNFα) were measured in two COVID-19 patient cohorts with high disease severity (ICUCovid; n=79) and with neurological complications (NeuroCovid; n=78), and in two control groups free from COVID-19 history, healthy subjects (n=20) and patients with amyotrophic lateral sclerosis (ALS; n=51). Samples from COVID-19 patients were collected during the first and the second wave of COVID-19 pandemic in Lombardy, Italy. Evaluations were done at acute and chronic phases of the COVID-19 infection.

**Results:**

Blood biomarkers of BBB disruption and neuronal damage are high in COVID-19 patients with levels similar to or higher than ALS. NeuroCovid patients display lower levels of the cytokine storm inducer PPIA but higher levels of MMP-9 than ICUCovid patients. There was evidence of different temporal dynamics in ICUCovid compared to NeuroCovid patients with PPIA and IL-10 showing the highest levels in ICUCovid patients at acute phase. On the contrary, MMP-9 was higher at acute phase in NeuroCovid patients, with a severity dependency in the long-term. We also found a clear severity dependency of NFL and GFAP levels, with deceased patients showing the highest levels.

**Discussion:**

The overall picture points to an increased risk for neurological complications in association with high levels of biomarkers of BBB disruption. Our observations may provide hints for therapeutic approaches mitigating BBB disruption to reduce the neurological damage in the acute phase and potential dysfunction in the long-term.

## Introduction

SARS-CoV-2 infection is associated with neurological symptoms and complications that range from headache, anosmia and dysgeusia, to severe complications such as cerebrovascular events, encephalopathy, Guillain-Barré syndrome, and dementia-like syndrome (1). In addition, many COVID-19 patients develop a ‘post-COVID-19 syndrome’ defined as the persistence of a wide spectrum of symptoms beyond four weeks after infection (2). In symptomatic COVID-19 patients, a community-based study with over half a million people in the UK estimated that about one in three experienced at least one persistent symptom for 12 weeks or more (3). In a population-based study in Lombardy, the post-COVID-19 condition was associated with death, rehospitalization and use of health resources (4). Long-term neuropsychological impairments such as executive, attentional and memory deficits, are reported even after mild infection (5). While the exact causes of post-COVID-19 syndrome remain largely elusive, the prevalence of associated neurological symptoms with an increased risk of anxiety and depression at 16-month follow-up (6) suggests a brain origin (7, 8).

There is neurochemical evidence of neuronal injury in patients with COVID-19 (9, 10), with reports of a severity-dependent increase of neurofilament light chain (NFL) at 4-month follow-up, further supporting ongoing brain injury even weeks and months after acute infection (11). Not surprisingly, the neurological complications are associated with worse functional outcome, particularly in older subjects and those with comorbidities (12).

Hypotheses of pathogenic processes implicated in acute and delayed brain injury following a SARS-CoV-2 infection include: i) viral invasion, ii) bioenergy failure, iii) autoimmunity, and iv) innate neuroimmune responses (13). In all these processes the blood-brain barrier (BBB), which maintains the specialized microenvironment of the neural tissue by regulating the trafficking of substances between the blood and brain compartments, has a central role.

Brain endothelial cells are the primary unit in close association with pericytes and astrocytes (14). Pericytes, which are key cells in maintaining and supporting vascular homeostasis and barrier function (15), are also the main source of matrix metalloproteinase 9 (MMP-9) (16, 17). Inflammatory stimuli very rapidly activate MMP-9 at the pericyte somata, leading to degradation of the underlying tight junction complexes. Thus, MMP-9 can act as a toxic culprit of BBB disruption after acute (18, 19) and neurodegenerative diseases (20). Peptidyl prolyl cis-trans isomerase A (PPIA), also known as cyclophilin A, acts as an activator of MMP-9 (21, 22) through binding to its CD147 receptor, which in addition has been proposed as an alternative route for SARS-CoV-2 infection (23).

Mechanistically, it has been demonstrated that the severe COVID-19-related cytokine storm is induced by a “spike protein-CD147-PPIA signaling axis” (24). *In vivo* experiments using a preclinical mouse model indicated that an anti-CD147 antibody inhibited the cytokine storm of SARS-CoV-2 (24).

Astrocytic end-feet containing glial fibrillary acidic protein (GFAP) are an essential component of the BBB. High blood GFAP is a marker of structural damage in the acute phase of brain injury and a severity-dependent increase has been detected in COVID-19 patients (11, 25). These data highlight PPIA, MMP-9, and GFAP as key disease biomarkers, so their measurement in association with NFL, an established marker of brain injury, may help identify a blood signature for disease severity and neurological complications in COVID-19 patients (NeuroCovid).

We identified significant effects associated with SARS-CoV-2 infection in COVID-19 patients, with NeuroCovid subjects showing the highest levels of biomarkers associated with BBB disruption, while patients in the intensive care unit (ICUCovid) had higher levels of inflammatory response biomarkers.

## Materials and methods

### Study approval

The study was approved by the ethics committees of the clinical centers involved: Fondazione IRCCS Ca’ Granda Ospedale Maggiore Policlinico, Milano (approval #868_2020, 28.10.2020), ASST Papa Giovanni XXIII, Bergamo (approval #123/20, 14.05.2020). Written consent was obtained from patients themselves or their legal representatives when they lacked capacity to consent. Wherever possible, informed consent was collected verbally. However, in most cases, due to the patient’s inability to provide informed consent or to collect it in compliance with the contagion prevention measures, the principle of secondary use of data was used in accordance with art. 28, paragraph 2, letter b) of the November 20, 2017 law, no. 167, included in the legislative decree 196/03 of art. 110-bis.

### Study populations

Two COVID-19 populations, referred to as ICUCovid and NeuroCovid, were recruited between February 2020 and February 2021. All participants received a positive PCR test for SARS-CoV-2 RNA on nasopharyngeal swab. Control groups free from COVID-19 history were patients with amyotrophic lateral sclerosis and a healthy population. Their main demographic and clinical characteristics are reported in **Table 1**.

**Table 1:** Demographic and clinical characteristics of the patient cohorts.

Characteristics	ICUCovid	NeuroCovid	ALS	Healthy
N	79	78	51	20
Age at sampling, years, median (IQR)	65 (58–70)	61 (53-71)	67 (62-71)	61 (58-63)
Sex (% males)	77%	73%	51%	35%
Hospitalization, days, median (IQR)	17 (10-28)	29 (10-51)	–	–
Mortality (% deceased)	34%	16%	–	–
PaO_2_/FIO_2_ ratio, median (IQR)	131 (93-180)	–	–	–
ALSFRS-R^1^ at sampling, median (IQR)	–	–	33 (22-38)	–

^1^ALSFRS-R: Revised Amyotrophic Lateral Sclerosis Functional Rating Scale.

#### ICUCovid

All patients admitted to the ICU, Rianimazione 1 Fiera Milano COVID-19 (Fondazione IRCCS Ca’ Granda Ospedale Maggiore Policlinico, Milan, Italy) were screened for eligibility. Inclusion criteria for this study population were: i) signed informed consent and ii) >18 years of age. Exclusion criteria were: i) known previous neurological conditions; ii) more than 48h in another ICU before admission; iii) pregnancy. Out of 296 screened patients, 79 were recruited for the study.

#### NeuroCovid

Patients admitted to the COVID-19 wards (ASST Papa Giovanni XXIII, Bergamo, Italy) with neurological manifestations confirmed by a neurological consultation/neurophysiological assessment/neuroradiologic investigation were recruited. Patients’ samples had been collected in an observational study on neurological manifestations in COVID-19 patients approved by the local Ethics Committee (257/2020, 13/5/2020) (26). The neurological diagnoses in this cohort are summarized in **Table 2** and included peripheral neuropathies (33% of patients), encephalopathies/encephalitis (33%) and cerebrovascular disorders (23%). Inclusion criteria were: i) signed informed consent; ii) > 18 years of age; iii) cognitive or neurological symptoms presenting during COVID-19 hospitalization, for which a neurological consultation/neurophysiological assessment/neuroradiologic investigation was required; iv) blood samples available. Of the 137 NeuroCovid patients, 78 fitted these criteria and their samples were included in the study. Patients were stratified based on clinical outcome: discharged fully recovered (moderate), discharged with sequalae (severe), and deceased (dead).

**Table 2:** Case definition for NeuroCovid cohort.

NeuroCovid total patients (N)	78
**Neurological complications**	N (%)
***Cerebrovascular disorders^1^***	*18 (23)*
Ischemic stroke	13
Hemorrhagic stroke	4
Transient ischemic attack	1
***Peripheral neuropathies***	*26 (33)*
Guillain Barrè Syndrome***^2^***	26
***Encephalopathies/Encephalites^3^***	*26 (33)*
***Miscellaneous***	*8 (10)*
Epilepsy	4
Myelopathy	1
Syncope	1
Movement disorder Headache	1 1

^1^As defined by Sacco et al. (27)

^2^As defined by Sejvar et al. (28)

^3^As defined by Quist-Paulsen et al. (29)

#### ALS patients and healthy controls

Informed written consent was obtained from all subjects involved and the study was approved by the ethics committee of Azienda Ospedaliero Universitaria Città della Salute e della Scienza, Turin. Healthy subjects and ALS patients had no COVID-19 history. The diagnosis of ALS was based on a detailed medical history and physical examination and confirmed by electrophysiological evaluation. Inclusion criteria for ALS patients were: i) >18 years old; ii) diagnosis of definite, probable or laboratory-supported probable ALS, according to revised El Escorial criteria. Exclusion criteria were: i) diabetes or severe inflammatory conditions; ii) active malignancy; iii) pregnancy or breast-feeding. ALS patients served as positive controls for severe neurodegeneration.

### Study design

**Figure 1:**
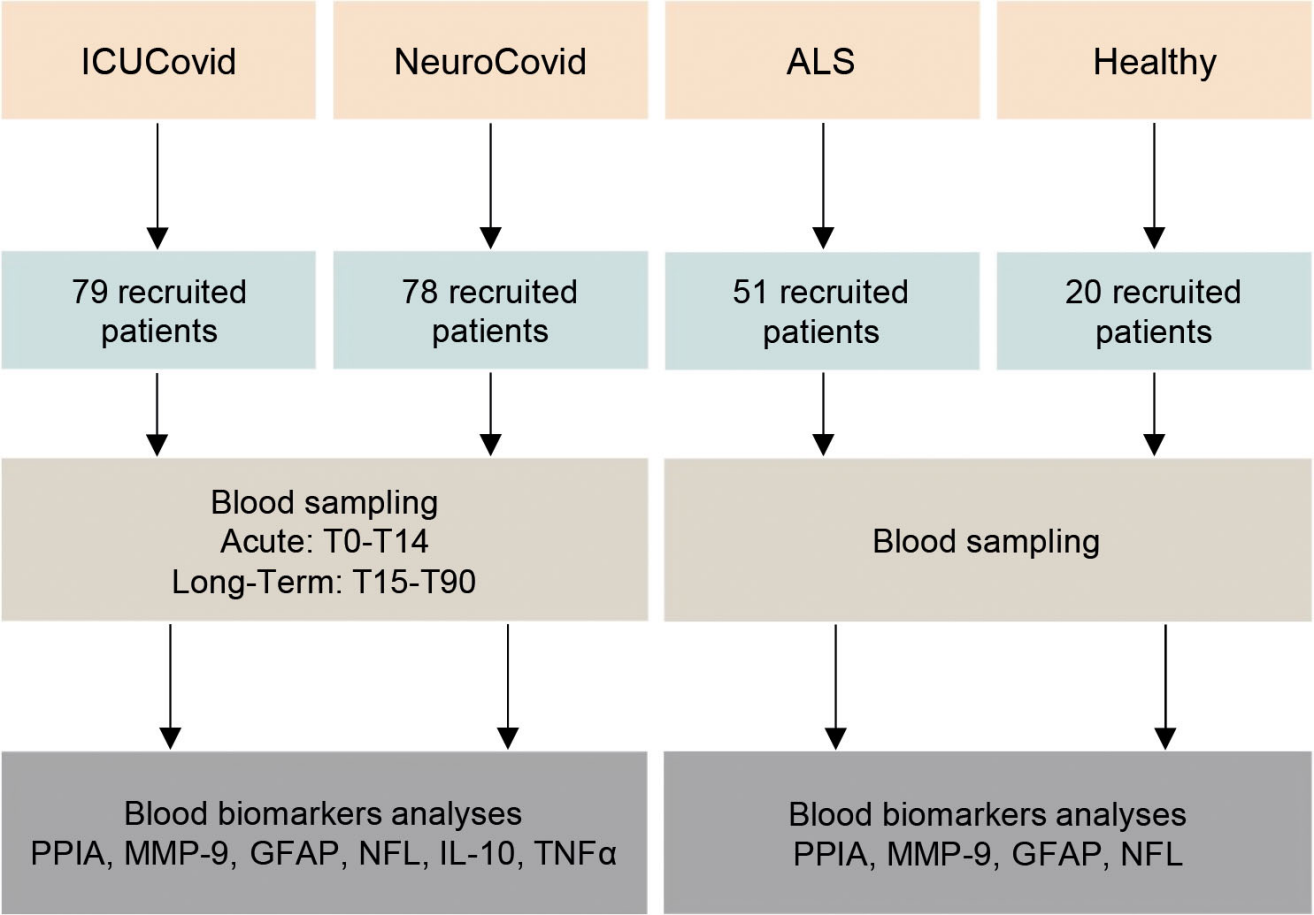
Schematic workflow for the biomarker characterization in two cohorts of COVID-19 patients and two cohorts of controls (ALS and healthy). The two cohorts of COVID-19 patients analyzed in the study are COVID-19 patients admitted to the ICU ward Rianimazione 1 Fiera Milano COVID-19 (ICUCovid; n=79) and to COVID-19 wards ASST Papa Giovanni XXIII, Bergamo, with neurological complications (NeuroCovid; n=78). Blood samples were drawn acutely at ICU admission and after 7-14 days (T0-T14), and in the long-term between 15 and 90 days in the ward (T15-T90). Plasma samples were isolated and then analyzed for PPIA, MMP-9, GFAP, NFL, IL-10 and TNFα biomarkers. Control groups were ALS patients (n=51) and healthy subjects (n=20). Plasma samples were isolated and then analyzed for PPIA, MMP-9, GFAP and NFL.

The study design is summarized in **Figure 1**. Two COVID-19 populations and ALS and healthy control groups were included (see *Study Populations* above). In the ICUCovid cohort, blood samples were drawn acutely at ICU admission (T0) and after 7 (T7) and 14 (T14) days. Clinical data were collected throughout the ICU stay and CT scans were done every two weeks when feasible. For the NeuroCovid cohort, the blood samples had initially been collected for clinical and not experimental purposes, so the samples available did not precisely match those collected in the ICUCovid cohort; therefore, we retrieved available samples from week 1 to week 2 in the ward (acute: T0-T14) and from longer timepoints (long-term: T15-T90). Clinical data were retrieved from medical records. Blood samples and clinical analyses were then done at the Istituto di Ricerche Farmacologiche Mario Negri IRCCS.

### Biomarker analysis

Bloods were processed at the contributing centers and plasma samples were aliquoted, cryopreserved at -80°C and shipped to the Istituto di Ricerche Farmacologiche Mario Negri IRCCS for biomarker analyses. Levels of NFL, GFAP, IL-10 and TNFα were measured using commercially available single molecule array assay kits on an SR-X Analyzer (Neuro 2-Plex B (#103520), interleukin-10 (IL-10) (#101643) and tumor necrosis factor (TNFα) (#101580) advantage kits) as described by the manufacturer (Quanterix, Billerica, MA). A single batch of reagents was used for each analyte. MMP-9 was measured with an AlphaLISA kit for the human protein (#AL3138, PerkinElmer). AlphaLISA signals were measured using an Ensight Multimode Plate Reader (PerkinElmer). PPIA was measured with an ELISA for the human protein (#RD191329200R, BioVendor).

### Other laboratory data

Clinical and outcome data were retrieved from medical records for all patients.

### Statistical analysis

For each variable the differences between experimental groups were analysed by a Mann Whitney test or Kruskall-Wallis test, followed by Dunn’s *post-hoc* tests. Two-way ANOVA for repeated measures followed by Sidak’s *post-hoc* test was used to analyse biomarkers in ICUCovid patients. *P* values below 0.05 were considered significant. Prism 8.0 (GraphPad Software Inc., San Diego, CA) was used.

### Data availability

All data produced in the present study are available upon reasonable request to the authors.

## Results

### Blood biomarkers of BBB disruption and neuronal damage are high in COVID-19 patients with levels similar to or higher than in a severe neurodegenerative disease

**Figure 2:**
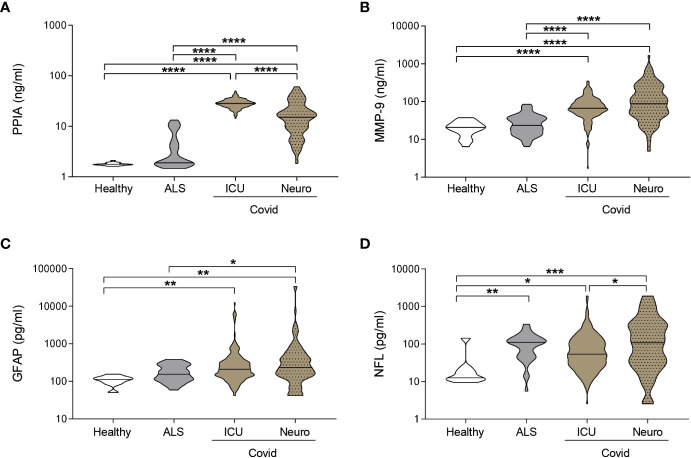
Biomarkers comparison between COVID-19 and a neurodegenerative disorder. **(A-D)** PPIA **(A)**, MMP-9 **(B)**, GFAP **(C)**, and NFL **(D)** concentrations were measured in plasma samples from ICUCovid patients (ICU n=79), NeuroCovid patients (n=78), ALS patients (PPIA n=50; MMP-9 n=51; GFAP and NFL n=34); and healthy controls (PPIA n=18; MMP-9 n=20; GFAP and NFL n=9). Violin plots indicate median, variability and probability density of biomarker concentrations. **(A, B, D)** Kruskal-Wallis, p < 0.0001; **(C)** Kruskal-Wallis, p = 0.0003. **(A-D)** *p < 0.05, **p < 0.01, ***p < 0.001, ****p < 0.0001 by Kruskal-Wallis, Dunn’s *post hoc* test.

PPIA, an inducer of MMP-9 (22) and cytokine storm (24), showed the highest levels in ICUCovid patients (**Figure 2A**), while MMP-9, which is strictly related to BBB disruption, is highest in NeuroCovid patients (**Figure 2B**). Both PPIA and MMP-9 in hospitalized COVID-19 patients are substantially higher than ALS patients, characterized by severe neurodegeneration, and healthy controls (**Figures 2A, B**). Plasma concentrations of GFAP were also high, irrespective of the neurological complications compared to healthy controls and were equal to or higher than the levels in ALS patients (**Figure 2C**). NFL has similar behavior, with the highest levels in NeuroCovid significantly higher than in ICUCovid patients (**Figure 2D**). These data suggest a clear neurological implication and call for a granular description of biomarker changes in these patient cohorts in relation to time and severity.

### NeuroCovid patients have lower levels of the cytokine storm inducer PPIA but higher levels of BBB disruption markers

**Figure 3:**
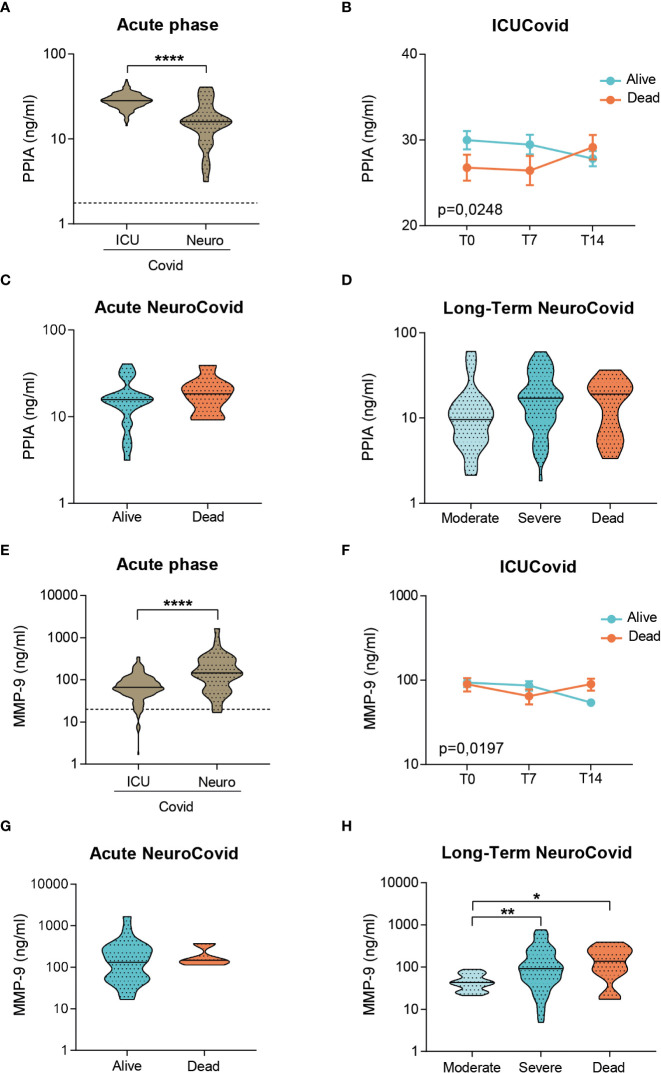
Analysis of PPIA and MMP-9 in plasma of two cohorts of COVID-19 patients. **(A-H)** PPIA **(A-D)** and MMP-9 **(E-H)** concentrations were measured respectively by ELISA and AlphaLISA technology in plasma samples from two cohorts of COVID-19 patients. **(A, E)** Violin plots indicate the median, variability and probability density of PPIA **(A)** and MMP-9 **(E)** at acute phase, in ICUCovid (n=79) and NeuroCovid samples (n=31). Dotted line indicates the mean level of healthy controls. **(A, E)** Mann Whitney, ****p < 0.0001. **(B, F)** The concentrations of PPIA **(B)** and MMP-9 **(F)** were measured in ICUCovid patients over time, at ICU admission (T0) and after 7 (T7) and 14 days (T14). ICUCovid patients were stratified as alive (n=32) and dead (n=14). Data (mean ± SEM) indicate biomarker concentrations. **(B)** Two-way ANOVA for repeated measures, p = 0.0248; **(F)** two-way ANOVA for repeated measures, p = 0.0197. **(C, G)** The concentrations of PPIA **(C)** and MMP-9 **(G)** were measured at acute phase, in samples from NeuroCovid patients, stratified as alive (n=23) and dead (n=8). Violin plots indicate median, variability and probability density of biomarker concentrations. **(C)** Mann Whitney, p = 0.3966; **(G)** Mann Whitney, p = 0.5498. **(D, H)** The concentrations of PPIA **(D)** and MMP-9 **(H)** in the long-term, in samples from NeuroCovid patients, stratified as moderate (n=18), severe (n=42) and dead (n=8). Violin plots indicate median, variability and probability density of biomarker concentrations. **(D)** Kruskal-Wallis, p = 0.1175. **(H)** Kruskal-Wallis, p = 0.0048; *p < 0.05, **p < 0.01 by Kruskal-Wallis, Dunn’s *post hoc* test.

We characterized the severity-dependent changes and temporal dynamics of PPIA, MMP-9, GFAP and NFL in ICUCovid and NeuroCovid patients. In the acute phase, ICUCovid patients had higher PPIA levels than NeuroCovid patients (**Figure 3A**). Among ICUCovid patients, a slight temporal increase was observed in the deceased group, leading to higher PPIA levels at 14 days than in alive patients (T14, **Figure 3B**). NeuroCovid patients showed no severity dependency in the acute and the longer phases (**Figures 3C, D**). In the acute phase, ICUCovid patients had lower MMP-9 levels than NeuroCovid patients (**Figure 3E**). In the ICUCovid cohort, there was a slight decrease over the first two weeks in alive patients, leading to significantly lower levels on day 14 compared to deceased patients (T14, **Figure 3F**). In the NeuroCovid cohort, MMP-9 levels were similarly high in alive and deceased patients in the acute phase, while in the longer term they showed severity dependency (**Figures 3G, H**).

**Figure 4:**
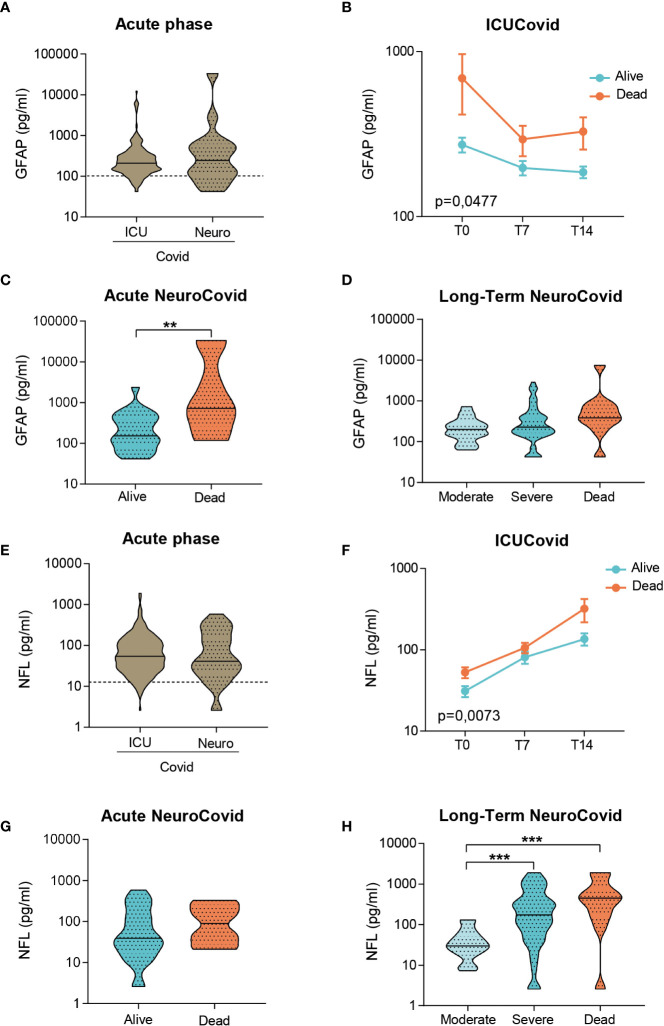
Analysis of GFAP and NFL in plasma of two cohorts of COVID-19 patients. **(A-H)** GFAP **(A-D)** and NFL **(E-H)** concentrations were measured by Simoa technology in plasma samples from two cohorts of COVID-19 patients. **(A, E)** Violin plots represent the median, variability and probability density of GFAP **(A)** and NFL **(E)** at acute phase, in ICUCovid (n=79) and NeuroCovid samples (n=31). Dotted line indicates the mean level of healthy controls. **(A)** Mann Whitney, p = 0.7910; **(E)** Mann Whitney, p = 0.7054. **(B, F)** The concentrations of GFAP **(B)** and NFL **(F)** were measured in ICUCovid patients over time, at ICU admission (T0) and after 7 (T7) and 14 days (T14). ICUCovid patients were stratified as alive (n=32) and dead (n=14). Data (mean ± SEM) indicate biomarker concentrations. **(B)** Two-way ANOVA for repeated measures, p = 0.0477; **p < 0.005 alive versus dead at T0 by Sidak’s *post hoc* test; **(F)** Two-way ANOVA for repeated measures, p = 0.0073; ***p < 0.001 alive versus dead at T14 by Sidak’s *post hoc* test. **(C, G)** The concentrations of GFAP **(C)** and NFL **(G)** were measured at acute phase, in samples from NeuroCovid patients, stratified as alive (n=23) and dead (n=8). Violin plots indicate median, variability and probability density of biomarker concentrations. **(C)** Mann Whitney, **p = 0.0088; **(G)** Mann Whitney, p = 0.2868. **(D, H)** The concentrations of GFAP **(D)** and NFL **(H)** in long-term samples from NeuroCovid patients, stratified as moderate (n=18), severe (n=42) and dead (n=8). Violin plots indicate median, variability and probability density of biomarker concentrations. **(D)** Kruskal-Wallis, p = 0.0570; **(H)** Kruskal-Wallis, p < 0.0001. ***p < 0.001 by Dunn’s *post hoc* test.

In the acute phase, GFAP levels did not differ between groups (**Figure 4A**). The longitudinal trajectories in ICUCovid patients showed an increase only in deceased patients, with the highest difference at admission (T0, **Figure 4B**). NeuroCovid patients displayed a high heterogeneity in GFAP levels in the acute phase (**Figure 4A**). This is due to GFAP severity dependency, significant in the acute phase and as a tendency in the long-term **(** **Figures 4C, D**).

In the acute phase, ICUCovid patients showed NFL levels like NeuroCovid patients (**Figure 4E**). The trajectories of live and death ICUCovid cohorts highlight a steep increase in NLF levels over the first two weeks, reaching the highest value for deceased patients at 14 days from ICU admission (T14, **Figure 4F**). While in the acute phase live and dead NeuroCovid patients have similar NFL levels **(** **Figure 4G**), in the long-term NFL levels showed a clear severity dependency (**Figure 4H**).

**Figure 5:**
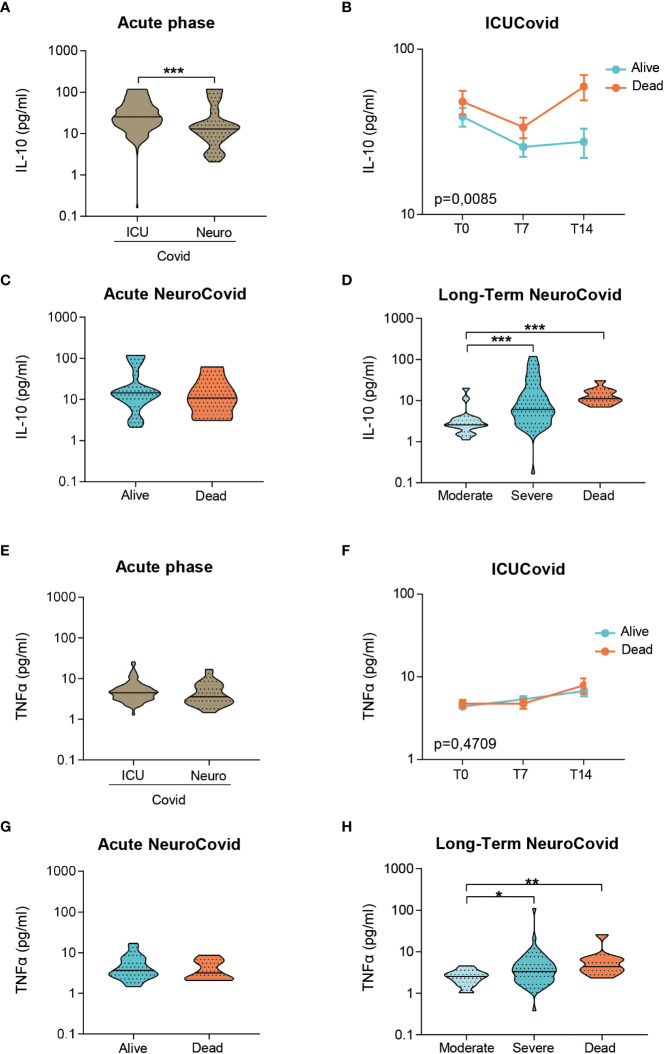
IL-10 and TNFα in plasma of two cohorts of COVID-19 patients. **(A-H)** IL-10 **(A-D)** and TNFα **(E-H)** concentrations were measured by Simoa technology in plasma from two cohorts of COVID-19 patients. **(A, E)** Violin plots of IL-10 **(A)** and TNFα **(E)** in the acute phase, in ICUCovid (n=79) and NeuroCovid samples (n=31). **(A)** Mann Whitney, ***p < 0.001. **(E)** Mann Whitney, p = 0.085. **(B, F)** IL-10 **(B)** and TNFα **(F)** were measured in ICUCovid patients at ICU admission (T0) and after 7 (T7) and 14 days (T14). ICUCovid patients were stratified as alive (n=32) or dead (n=14). Data (mean ± SEM) indicate biomarker concentrations. **(B)** Two-way ANOVA for repeated measures, p < 0.01 for cohort factor; **p < 0.005 alive versus dead at T14 by Sidak’s *post hoc* test. **(F)** Two-way ANOVA for repeated measures, p = 0.4709. **(C, G)** IL-10 **(C)** and TNFα **(G)** were measured in the acute phase in samples from NeuroCovid patients, stratified as alive (n=23) or dead (n=8). **(C)** Mann Whitney, p = 0.6652; **(G)** Mann Whitney, p = 0.5498. **(D, H)** The concentrations of IL-10 **(D)** and TNFα **(H)** at a longer time, in samples from NeuroCovid patients, stratified as moderate (n=18), severe (n=42) or dead (n=8). **(D)** Kruskal-Wallis, p < 0.0001; ***p < 0.001 by Kruskal-Wallis, Dunn’s *post hoc* test. **(H)** Kruskal-Wallis, p < 0.01; *p < 0.05, **p < 0.01 by Kruskal-Wallis, Dunn’s *post hoc* test.

Inflammatory markers of systemic immune response, including IL-10 and TNFα, were also measured. In the acute phase, IL-10 levels were highest in ICUCovid compared to NeuroCovid patients (**Figure 5A****),** with a clear increase in ICUCovid deceased patients at day 14 (T14, **Figure 5B****)**. Within the NeuroCovid cohort, IL-10 levels were similar in alive and deceased patients (**Figure 5C****)**. In the long-term, however, severity dependency was observed (**Figure 5D****)**.

In the acute phase, TNFα levels did not differ between ICUCovid and NeuroCovid patients (**Figure 5E****)** and within ICUCovid cohort there were no temporal changes in alive and deceased patients up to day 14 (T14, **Figure 5F****).** In the NeuroCovid cohort, acute TNFα levels were similar in alive and deceased patients **(** **Figure 5G**). In the long-term, however, NeuroCovid patients showed a significant severity dependency (**Figure 5H**).

## Discussion

This study examined the effects of SARS-CoV-2 infection on blood biomarkers of BBB disruption, neuronal damage and systemic inflammation by longitudinally monitoring two patient cohorts of COVID-19, with increasing disease severity and neurological complications. Blood biomarkers of BBB disruption were elevated in COVID-19 patients with levels comparable to or even higher than in ALS patients, pointing to neurological implications over a range of disease severities.

**Figure 6:**
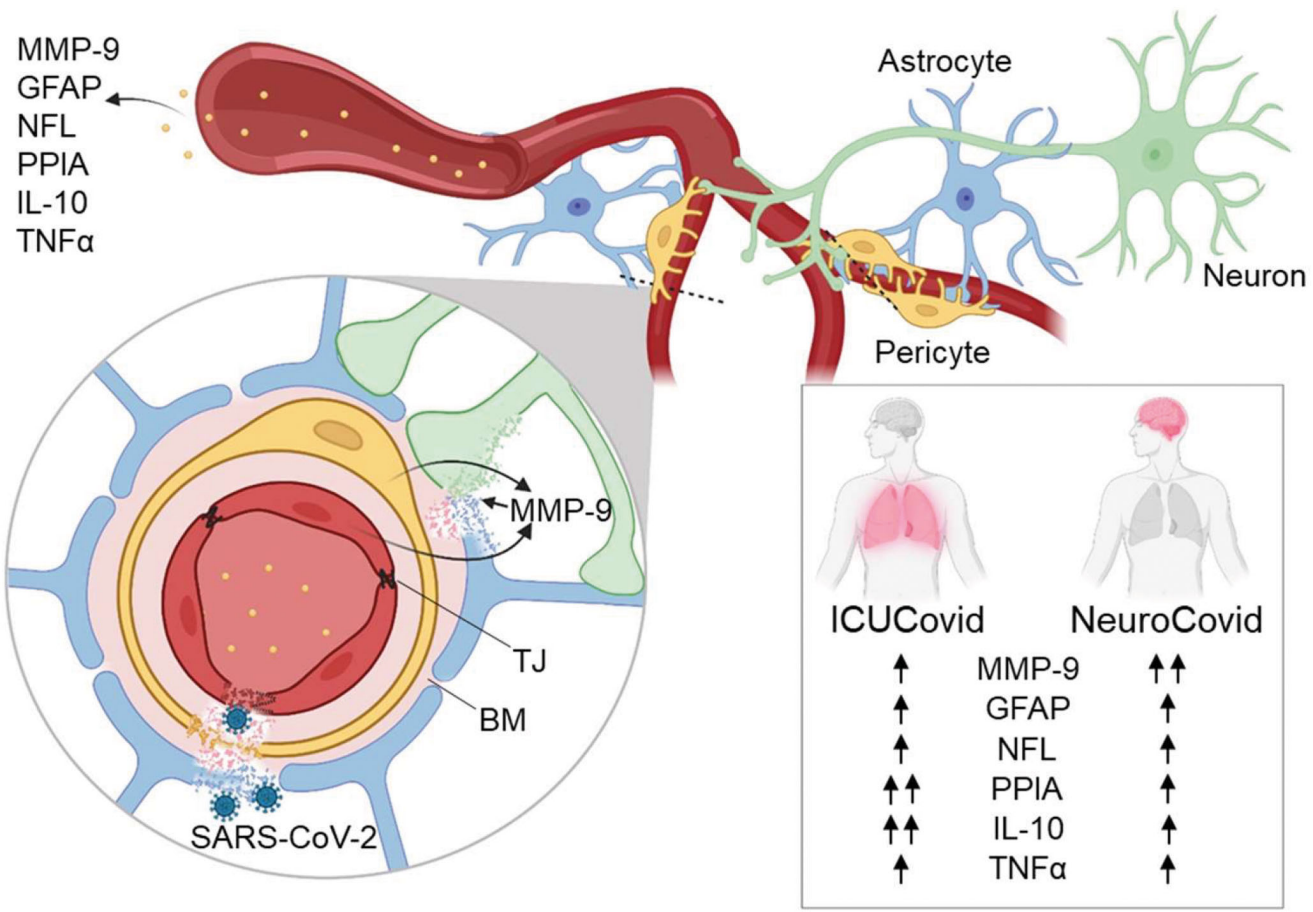
Highlights of the results. The effect of SARS-CoV-2 infection on blood biomarkers of BBB disruption (MMP-9, GFAP), neuronal damage (NFL) and systemic inflammation (PPIA, IL-10, TNFα) was measured in patient cohorts with high disease severity (ICUCovid) and with neurological complications (NeuroCovid). There were higher levels of PPIA and IL-10 in ICU compared to NeuroCovid patients, while MMP-9 was significantly higher in NeuroCovid patients. Over-activation of MMP-9 may lead to degradation of tight junctions (TJ), basement membrane (BM) and laminin, implying BBB disruption, penetration of SARS-CoV-2 into the brain and neuronal damage. Blood biomarkers of BBB disruption and neuronal damage were elevated in all COVID-19 patients suggesting potential neurological dysfunctions in the long-term, over a range of disease severities. Figure created with BioRender.com.

There was evidence of different temporal dynamics in ICUCovid compared to NeuroCovid patients with PPIA, the potent activator of the cytokine storm and MMP-9 inducer, and IL-10, the master regulator of immunity to infection, with the highest levels in ICUCovid patients in the acute phase (**Figure 6**). In contrast, MMP-9 was significantly higher in the acute phase in NeuroCovid patients, with severity dependency in the longer term. In line with previous findings, we found also clear severity dependency of NFL and GFAP levels with the highest levels in deceased patients, and severe NeuroCovid patients showing a tendency to maintain higher values than moderate patients in the longer term.

PPIA is a foldase and a molecular chaperone with multiple functions and substrates, including viral proteins essential for coronavirus replication (30). PPIA is a major target of redox regulation in activated lymphocytes (31, 32). Under stress conditions PPIA is secreted extracellularly by several types of cells, including pericytes, vascular smooth muscle cells and macrophages, behaving as a pro-inflammatory cytokine, with potent chemotactic activity toward leukocytes (21, 33, 34). Through the interaction with its CD147 receptor, in a NF-κB-dependent pathway, PPIA is an inducer of MMP-9 and of pro-inflammatory cytokines and chemokines (22, 35). High levels of PPIA have been seen in biofluids of several conditions associated with inflammation, including neurological and cardiovascular diseases (22, 36). Interestingly, high plasma concentrations of PPIA have also recently been reported in COVID-19 patients with mechanistic evidence for its involvement in the induction of the cytokine storm by activating CD147 (24).

A growing body of clinical data suggests that the cytokine storm is associated with COVID-19 severity, ICU admission, and is a crucial cause of death (37). In agreement with this, our ICUCovid patients had PPIA concentrations substantially higher than NeuroCovid patients. Also noteworthy is the extremely high PPIA plasma concentration in all COVID-19 patients. This may be linked to its up-regulation upon interaction of SARS-CoV-2 with CD147, as observed in animal models (24), and may favor viral replication (30, 38). Similarly, MMP-9 was very high in all COVID-19 patients. However, NeuroCovid patients had the highest levels of MMP-9 in the acute phase, with persistent high levels in most severe patients in the long-term. MMP-9 is a metalloproteinase with a wide substrate spectrum and is an important mechanism for fine-tuning cellular processes, but if aberrantly activated it is a key factor in BBB disruption and neuronal damage, by degrading tight junction proteins and laminin (18–20). MMP-9 can be induced by inflammatory signaling cascades with CD147 acting as the major upstream inducer in the CNS (39). CD147 is highly expressed in the brain capillary endothelium and various sub-regions of the brain (40). Brain pericytes are the main source of MMP-9 at the neurovascular unit and it is rapidly released in response to inflammatory stimuli (16, 17). It has also been demonstrated *in vitro* and *in vivo* that SARS-CoV-2 can infect the brain microvascular endothelial cells and cross the BBB by MMP-9-mediated disruption of basement membrane (41). Therefore, one can hypothesize that a local, early high MMP-9 concentration at the neurovascular unit in NeuroCovid patients, rather than extensive systemic inflammation as in ICUCovid patients, may be responsible for the BBB disruption that triggers neurological complications following SARS-CoV-2 infection.

Astrocytic end feet cover more than 99% of the neurovascular surface and directly affect BBB permeability (42). GFAP is a highly expressed protein of the CNS, almost exclusively in astrocytes. In neuropathological conditions, GFAP is released into the bloodstream either by direct venous drainage or through a compromised BBB (43). Blood GFAP can therefore serve as a useful biomarker and prognostic tool for numerous neurological conditions (25).

While classically considered a marker of astrogliosis, the presence of glial-derived proteins in peripheral body fluids has been suggested as indicating BBB disruption in acute CNS injury (44). In the case of traumatic brain injury, it has been recently suggested that high blood GFAP concentrations might reflect damage to astrocytic end feet enveloping the BBB, thus releasing GFAP directly into the blood when the BBB is injured (45). Elevated GFAP plasma levels have been reported in COVID-19 patients (10, 45) and were in line with neuropathological data indicating post-mortem evidence of BBB disruption and gliosis (46). In accordance with this, here we report high GFAP levels in a severity-dependent manner, with significantly higher levels at acute timepoints in deceased patients. GFAP only tended to be higher in NeuroCovid patients than in ICUCovid patients. However, the NeuroCovid cohort included several patients with Guillain-Barré syndrome in which blood-nerve-barrier (BNB) disruption is a key step (47). BNB lacks astrocytes and glia limitans, so the detection of barrier damage through GFAP in these cases is underestimated. Maladaptive microglia and monocyte activation may also exert a detrimental effect on BBB function and integrity in COVID-19 (14). Interestingly, a recent publication has shown that microglia−derived chemokine MCP-1 (also known as CCL2) seems to have a major role in neocortex neuroinflammation and BBB disruption in a mouse model of autoimmune encephalomyelitis (48). Moreover, high blood MCP-1 levels have been associated with disease severity and mortality in COVID-19 (49). Although not measured in our study, longitudinal analyses of MCP-1 in plasma and CSF of COVID-19 patients and correlation with neurological symptoms will shed light on this aspect in future studies.

NFL is an established marker of axonal injury (50). Although axonal degeneration is not a specific feature of ALS, NFL is considered its most characteristic biomarker since its concentration is higher than in any other neurological disease (51, 52). This may be because neurofilaments are abundantly expressed in the large myelinated axons involved in the degenerative process, which is particularly fast and severe in ALS compared to other diseases. The only other condition in which the NFL plasma concentration is as high as in ALS is HIV-associated dementia (HAD) (53). Interestingly, it seems that HIV-related CNS degeneration starts during primary infection and continues during subsequent stages of the disease. However, CSF NFL levels in primary infection are associated with CNS immune activation and BBB disruption but are not accompanied by high CSF total tau and low amyloid beta peptides, as in subjects with HAD (54). This indicates that this early neuronal injury is less severe and/or involves a different mechanism and can in fact be halted by antiretroviral therapy (55). Plasma NFL levels were high in all COVID-19 patients, with NeuroCovid patients reaching the same high levels as in HAD (53). Although the overall picture points to an increased risk for neurological dysfunctions in the long-term, the mechanism and extent to which acute axonal damage, in combination with systemic inflammation and BBB disruption, can predispose to neurodegeneration calls for further investigation.

Our observations may provide hints for a preventive approach. Should further evidence confirm that the neuronal damage found is secondary to, or exacerbated by, BBB disruption, therapies reducing BBB damage could serve as a valuable aid in attenuating the neurological damage in the acute phase and potential dysfunction in the longer term. Interestingly, MMP-9 stands as a druggable target since a set of potent MMPs inhibitors are already available for clinical use (56), furthermore drugs targeting the PPIA-CD147-MMP-9 signaling pathway are also under investigation. A PPIA inhibitor, cyclosporine A (CsA), a well-known immunosuppressive drug, and Meplazumab, an anti-CD147 monoclonal antibody, are being assessed in clinical trials up to phase 2/3 (NCT05113784) for the treatment of severe COVID-19 (57). There are some indications from observational studies of milder COVID-19 and lower mortality in solid organ transplant recipients and autoimmune disease patients under CsA treatment (58). Last, there is evidence that Annexin A1, an endogenous molecule endowed with resolving/protecting action on tight junctions, may have therapeutic potential in restoring cerebrovascular damage and BBB disruption in neurodegenerative diseases and metabolic disorders (59). Indeed, human recombinant annexin A1 has been recently shown to restore BBB integrity and reduce the expression and activity of MMP-9 in brain microvessels when administered in an experimental model of metabolic diabetic disorder (60). Thus, also Annexin A1 could be a therapeutic avenue for COVID-19 to explore in future studies.

There are limitations in this study that should be highlighted. First, neurocognitive assessment in these cohorts of patients was not performed, thus the question as to whether BBB biomarker changes may predict late cognitive dysfunction is still open and should be addressed in future studies. 13% NeuroCOVID patients had a known history of mild cognitive impairment possibly contributing to the altered neurologic state observed in the acute phase. However, in these patients acute NFL levels were comparable to or even lower than the other patients in the NeuroCovid group, making it unlikely that an already altered CNS homeostasis was the cause of the biomarker changes. Notably, in the ICU cohort there were no patients with pre-existing neurological conditions thus reinforcing the finding that COVID-19 *per se* may induce markers of BBB disruption and neurological damage. Last, patients in our study were recruited before the vaccination campaign. Although there is increasing evidence that COVID-19 vaccination may have a protective effect against the post-COVID-19 syndrome (61, 62), this aspect has not been fully explored, calling for follow-up studies to monitor distinct long-term consequences in vaccinated and non-vaccinated subjects.

Despite these caveats, our study may provide hints for upcoming therapeutic approaches for COVID-19 mitigating at the same time BBB disruption and neurodegeneration to reduce the neurological damage in the acute phase and potential dysfunction in the long-term.

## Data availability statement

The original contributions presented in the study are included in the article. Further inquiries can be directed to the corresponding authors.

## Ethics statement

The studies involving human participants were reviewed and approved by the ethics committees of the clinical centers involved: Fondazione IRCCS Ca’ Granda Ospedale Maggiore Policlinico, Milano (approval #868_2020, 28.10.2020), ASST Papa Giovanni XXIII, Bergamo (approval #123/20, 14.05.2020). The patients/participants provided their written informed consent to participate in this study.

## Author contributions

VB, NS, MS, EZ: Drafting/revision of the manuscript, major role in the acquisition of data, study concept and design, analysis and interpretation of data. LP, IL: Drafting/revision of the manuscript, major role in the acquisition of data, analysis and interpretation of data. MC, NB: Drafting/revision of the manuscript, study concept and design. RZ, EF, VP, SL, GC, MG, NR, AC, CM: Drafting/revision of the manuscript, major role in the acquisition of data. BB, GF, RG, FBl, CC, NM, MT, PB, FBi, AB, AN: Drafting/revision of the manuscript. All authors contributed to the article and approved the submitted version.
